# Personalized Breast Reconstruction After Breast-Conserving Therapy: Risk-Informed Approaches to Technique Selection and Timing

**DOI:** 10.3390/jpm16040197

**Published:** 2026-04-01

**Authors:** Thomas J. Sorenson, Carter J. Boyd, Rebecca Lisk, Nolan S. Karp

**Affiliations:** Hansjorg Wyss Department of Plastic Surgery, NYU-Langone Health, New York, NY 10016, USA; thomas.sorenson@nyulangone.org (T.J.S.); rebecca.lisk@nyulangone.org (R.L.)

**Keywords:** breast conserving therapy, breast reconstruction, patient reported outcomes, aesthetic, personalized medicine

## Abstract

Breast-conserving therapy (BCT), consisting of lumpectomy followed by adjuvant radiation, provides oncologic outcomes equivalent to mastectomy for many patients with breast cancer. As survivorship increases, the demand for aesthetic restoration after BCT has grown; however, reconstructive strategies in this setting remain less standardized than those following mastectomy. Reconstruction after BCT presents distinct challenges due to partial tissue loss, nonuniform radiation injury, progressive fibrosis, and wide variability in patient expectations and tolerance for revision surgery. Consequently, mastectomy-based reconstructive algorithms are often insufficient for guiding care in this population. This review synthesizes contemporary reconstructive options following BCT through a personalized medicine framework, emphasizing patient-specific risk factors that influence technique selection, timing, and long-term outcomes. Key determinants include radiation exposure, breast morphology, comorbid conditions, prior breast surgery, and psychosocial preferences. Oncoplastic volume displacement, implant-based augmentation, fat grafting, and autologous reconstruction each demonstrate distinct risk profiles in the post-BCT tissue environment and require individualized application. Timing of reconstruction and willingness to undergo staged procedures play a central role in outcome durability and patient satisfaction. Across reconstructive strategies, revision burden emerges as a clinically meaningful, patient-centered outcome that is not adequately captured by traditional short-term complication metrics. A risk-informed approach that integrates individualized risk assessment with transparent counseling and shared decision-making may improve alignment between reconstructive planning and patient goals. Personalized reconstruction after BCT requires moving beyond technique-driven paradigms toward flexible, longitudinal care pathways. Future efforts should focus on developing BCT-specific predictive models and incorporating patient-reported outcomes to advance personalized reconstructive care.

## 1. Introduction

Breast-conserving therapy (BCT), consisting of lumpectomy followed by adjuvant radiation, has become a cornerstone of contemporary breast cancer management, offering oncologic outcomes equivalent to mastectomy for appropriately selected patients while preserving native breast tissue [1,2]. As long-term survival has improved, increasing attention has been directed toward the aesthetic and psychosocial sequelae of BCT [3,4,5,6]. Despite the widespread adoption of breast conservation, reconstructive strategies for patients who experience contour deformity, volume asymmetry, or radiation-induced tissue changes after lumpectomy remain far less standardized than those following mastectomy [7].

Reconstruction after BCT presents a distinct clinical challenge that differs fundamentally from post-mastectomy reconstruction. Unlike mastectomy patients, individuals treated with lumpectomy retain variable amounts of breast tissue that have often been exposed to nonuniform radiation fields, resulting in fibrosis, volume contraction, and compromised vascularity. These changes create a heterogeneous tissue environment in which reconstructive outcomes are less predictable, and complication profiles vary widely [8,9]. Moreover, reconstructive interventions in this setting must account for ongoing oncologic surveillance, patient tolerance for asymmetry or staged procedures, and the cumulative burden of revision surgery over time [10].

Current reconstructive paradigms are largely derived from mastectomy-based algorithms and are frequently applied to BCT patients without sufficient consideration of their unique risk profiles. Implant-based and fat grafting techniques following lumpectomy are often discussed in isolation, with limited integration of patient-specific factors such as radiation exposure, breast morphology, comorbidities, and prior surgical history [11,12,13]. As a result, counseling may inadequately reflect the true likelihood of complications, revision procedures, or delayed aesthetic dissatisfaction in this population.

Personalized medicine offers a framework through which reconstruction after BCT can be more effectively tailored to the individual patient. Rather than emphasizing a single “best” reconstructive approach, personalization prioritizes risk information, patient preferences, and contextual decision-making to guide technique selection and timing. In this model, outcomes such as revision burden, durability of aesthetic results, and patient-reported satisfaction become central metrics of success, complementing traditional complication rates.

This review aims to synthesize the existing literature on reconstructive options following breast-conserving therapy through a personalized medicine lens. We focus on patient-specific risk factors that influence reconstructive outcomes, examine how these risks intersect with available reconstructive techniques, and propose a risk-informed framework to guide individualized decision-making. By integrating oncologic considerations, tissue characteristics, and patient goals, this review seeks to provide a practical and personalized approach to reconstruction after BCT that better aligns surgical planning with long-term outcomes and patient expectations.

## 2. Literature Review Approach

This narrative review synthesizes current literature addressing reconstructive strategies following breast-conserving therapy (BCT). A targeted literature search was performed using PubMed/MEDLINE, Embase, and Google Scholar to identify relevant studies evaluating partial breast reconstruction techniques, oncoplastic surgery, implant-based augmentation, autologous fat grafting, and autologous reconstruction in the post-BCT setting. Search terms included combinations of “breast-conserving therapy,” “partial breast reconstruction,” “oncoplastic surgery,” “fat grafting,” “implant augmentation after lumpectomy,” and “breast reconstruction after radiation.”

Priority was given to recent systematic reviews, prospective studies, large retrospective cohorts, and landmark publications relevant to reconstructive decision-making after BCT. Articles were selected based on relevance to reconstructive technique selection, patient risk factors, timing of reconstruction, and patient-reported outcomes. The literature reviewed primarily spans studies published from 2000 through 2025, with earlier foundational studies included where appropriate.

## 3. Unique Challenges After Breast-Conserving Therapy

Reconstruction following breast-conserving therapy (BCT) occurs within a biologically and anatomically altered tissue environment that differs substantially from that encountered after mastectomy. The combination of partial tissue excision and adjuvant radiation produces variable changes in breast volume, contour, and skin quality, resulting in reconstructive challenges that can be underestimated [14,15]. Unlike mastectomy reconstruction, where the surgical field is relatively uniform and reconstruction replaces the entirety of the breast mound, post-BCT reconstruction must integrate new volume or contour correction into a variably asymmetric and potentially fibrotic, scarred breast.

### 3.1. Altered Tissue Biology After Lumpectomy and Radiation

Lumpectomy creates a localized volume deficit that may evolve over time as postoperative changes and radiation effects progress. Adjuvant radiation further compounds these changes by inducing fibrosis, microvascular injury, and chronic inflammation, leading to progressive tissue stiffening, skin thickening, and volume contraction [16]. Importantly, radiation fields following lumpectomy are often nonuniform, resulting in asymmetric tissue injury that can affect reconstructive predictability. This heterogeneity in tissue quality increases the risk of wound healing complications, fat necrosis, capsular contracture, and suboptimal aesthetic outcomes, particularly when implant-based or volume replacement techniques are employed [17,18].

The temporal nature of radiation-induced changes adds another layer of complexity. Tissue characteristics may continue to evolve for years after treatment, meaning that reconstructive outcomes achieved early may not be durable over time [19]. This dynamic process complicates both surgical planning and patient counseling, as the final aesthetic result may not stabilize until long after reconstruction has been completed.

### 3.2. Oncologic and Surveillance Considerations

Reconstructive decision-making after BCT must also remain closely aligned with oncologic priorities. Margin status, tumor biology, and the need for ongoing surveillance imaging can influence both the timing and type of reconstruction offered [14]. Concerns regarding interference with mammographic or magnetic resonance imaging surveillance persist, particularly with implant-based augmentation or extensive fat grafting. While contemporary imaging techniques have largely mitigated these issues, lingering uncertainty may influence patient and provider comfort with certain reconstructive approaches [20,21].

Additionally, reconstructive interventions following BCT must account for the possibility of future oncologic events, including ipsilateral recurrence or the need for completion mastectomy [22]. Reconstructive strategies that limit future options or increase surgical complexity in the event of recurrence may not be appropriate for all patients, underscoring the importance of individualized risk assessment [14].

### 3.3. Aesthetic Expectations and Asymmetry Tolerance

Patients undergoing reconstruction after BCT often seek restoration rather than replacement of the breast, with expectations that their postoperative appearance will approximate their pre-treatment baseline. However, tolerance for residual asymmetry varies widely and is influenced by breast size, pre-existing asymmetry, clothing preferences, and psychosocial factors [23]. Unlike mastectomy reconstruction, where bilateral symmetry can often be addressed concurrently, post-BCT reconstruction may require contralateral surgery, staged procedures, or acceptance of persistent asymmetry.

These considerations make reconstruction after BCT particularly sensitive to patient expectations and risk tolerance. Some patients may prioritize minimal intervention and accept modest asymmetry, while others may pursue multiple revision procedures to achieve a desired aesthetic outcome. Failure to align reconstructive strategy with these individualized preferences can lead to dissatisfaction even in the absence of surgical complications, highlighting the importance of appropriate preoperative counseling [24].

### 3.4. Revision Burden as a Distinct Outcome Metric

A defining feature of reconstruction after BCT is the cumulative burden of revision surgery [8]. Many reconstructive approaches in this setting, particularly fat grafting and implant augmentation, are inherently staged and may require multiple procedures to achieve or maintain acceptable outcomes. Radiation-related changes, delayed volume loss, and progressive fibrosis further contribute to the need for secondary interventions [25].

Traditional outcome reporting, which often focuses on short-term complication rates, inadequately captures this longitudinal experience [26,27]. For BCT patients, revision burden represents a clinically meaningful and patient-centered outcome that must be incorporated into reconstructive planning and counseling [8,23]. Recognizing revision likelihood as a function of patient-specific risk factors provides a foundation for a more personalized and transparent approach to reconstruction.

## 4. Patient Factors Driving Personalized Reconstruction After Breast Conserving Therapy

Reconstruction following breast-conserving therapy (BCT) is uniquely sensitive to patient-level variability (Table 1). Unlike mastectomy reconstruction, where standardized pathways can often be applied, outcomes after BCT are heavily influenced by a constellation of demographic, treatment-related, anatomic, and psychosocial factors [3,28,29,30]. Appreciating how these variables interact is essential for tailoring reconstructive strategy, counseling patients effectively, and anticipating both complications and revision burden.

### 4.1. Demographic and Medical Factors

Patient comorbidities play a central role in determining reconstructive risk after BCT, particularly in the setting of irradiated tissue. Elevated body mass index, smoking history, diabetes, and connective tissue disease have all been associated with impaired wound healing, increased rates of infection, and compromised aesthetic outcomes [29,31,32,33]. In the post-BCT population, these risks are amplified by radiation-induced microvascular injury and fibrosis, which limit tissue reserve and reduce tolerance for additional surgical insult [34,35,36,37]. Age may also influence reconstructive decision-making, not only through physiologic considerations but through differences in aesthetic priorities, willingness to undergo staged procedures, and tolerance for asymmetry [38,39]. Importantly, these demographic factors rarely operate in isolation; rather, they compound one another, underscoring the need for holistic risk assessment rather than single-variable exclusion criteria [40].

### 4.2. Treatment-Related Factors

Among all patient-specific variables, prior radiation exposure exerts the most profound and consistent influence on reconstructive outcomes after BCT [41]. Radiation dose, fractionation, and field geometry affect both the severity and distribution of tissue injury [42,43]. Nonuniform radiation fields following lumpectomy create asymmetric patterns of fibrosis and volume loss that complicate reconstruction and reduce predictability, particularly for implant-based approaches [44]. The timing of reconstruction relative to radiation is another critical determinant of risk. Early intervention may occur before radiation effects have fully declared themselves, whereas delayed reconstruction must contend with established fibrosis and tissue contraction [44]. Chemotherapy, particularly when administered concurrently or sequentially with radiation, may further impair wound healing and influence the optimal timing of reconstruction [45,46]. These factors highlight the importance of individualized timing decisions rather than strict adherence to dogma.

### 4.3. Breast-Specific and Anatomic Factors

Breast morphology strongly influences reconstructive options after BCT. Breast size, degree of ptosis, and baseline asymmetry affect the feasibility of volume displacement techniques and the likelihood that contralateral symmetrization will be required [15,28,47,48]. Smaller breasts with limited tissue redundancy may be less forgiving of lumpectomy-related volume loss and may necessitate volume replacement strategies, whereas larger breasts may better accommodate oncoplastic rearrangement [17].

Tumor location also has important reconstructive implications. Defects in the lower pole or central breast often produce more visible deformities and are less amenable to simple rearrangement, while upper pole defects may be more easily concealed [29,49]. Prior breast surgery, including augmentation or reduction, introduces additional complexity by altering tissue planes, vascularity, and implant–tissue interactions [50]. Patients with pre-existing implants represent a distinct subgroup in whom implant-based augmentation after lumpectomy may be appealing but carries unique risks related to capsular contracture and implant malposition in the setting of radiation [51].

### 4.4. Patient Preferences and Psychosocial Factors

Personalized reconstruction after BCT must extend beyond physiologic risk to encompass patient values, expectations, and tolerance for trade-offs. Patients vary widely in their willingness to undergo staged procedures, accept temporary or permanent asymmetry, or assume the risks associated with implants or fat grafting [52]. Some prioritize minimal intervention and rapid recovery, while others are motivated to pursue multiple revisions to achieve a specific aesthetic outcome. Psychosocial context, including body image, professional and lifestyle demands, and prior experiences with cancer treatment, shapes these preferences and influences satisfaction independently of objective surgical outcomes [53,54,55]. Failure to account for these factors can result in discordance between surgeon-defined success and patient-reported satisfaction.

A critical but underappreciated dimension of risk after BCT reconstruction is the cumulative burden of revision surgery [27]. Many reconstructive strategies in this setting are inherently iterative, with outcomes evolving over time as radiation effects progress and tissue characteristics change [8,19]. Fat grafting often requires multiple sessions, while implant-based augmentation may necessitate revisions for capsular contracture, asymmetry, or contour deformity [56,57]. Traditional complication metrics fail to capture this longitudinal experience. For patients, the number, timing, and impact of revision procedures represent meaningful outcomes that influence quality of life, satisfaction, and overall perception of reconstruction success [58]. Incorporating anticipated revision burden into preoperative counseling allows for more transparent decision-making and aligns reconstructive strategy with patient-specific risk tolerance.

## 5. Personalized Decision-Making After Breast-Conserving Therapy: Risk-Informed Reconstructive Options

**Figure 1:**
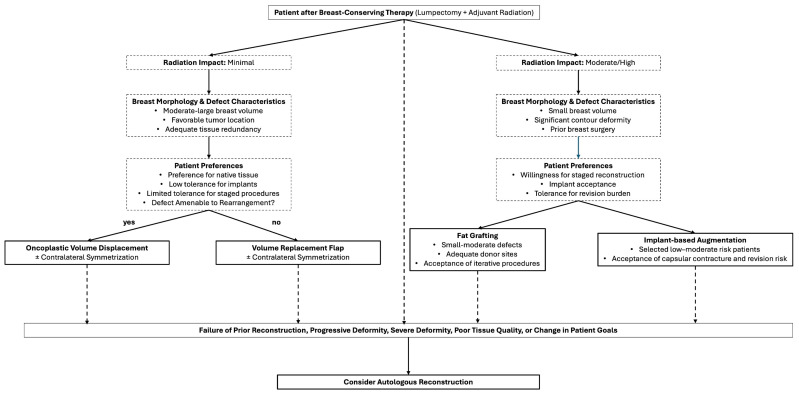
Conceptual framework for personalized reconstruction after breast-conserving therapy. Reconstructive options are presented as complementary strategies that may be applied sequentially or selectively depending on patient-specific risk factors, tissue characteristics, and reconstructive goals. Autologous reconstruction may be considered either as a primary approach in patients with severe deformity or compromised tissue quality or as a salvage option following prior reconstructive interventions.

Reconstructive decision-making after breast-conserving therapy (BCT) is best conceptualized as a continuum of options rather than a discrete set of interchangeable techniques. Patient-specific risk factors, including tissue quality, radiation exposure, breast morphology, and tolerance for revision, should guide both the selection and sequencing of reconstructive interventions [14,15,47,59,60]. A risk-informed framework allows surgeons to match reconstructive intensity to patient context, balancing aesthetic goals against the likelihood of complications and revision burden (Figure 1) [15].

Given the relative scarcity of prospective studies specifically addressing reconstruction after breast-conserving therapy, many recommendations in this field are informed by retrospective data, expert consensus, and extrapolation from broader breast reconstruction literature. It is important to note that this framework is conceptual and intended to illustrate how patient-, treatment-, and tissue-specific factors may be integrated into reconstructive decision-making. The model reflects synthesis of existing literature and clinical experience rather than a prospectively validated risk prediction algorithm.

### 5.1. Oncoplastic Volume Displacement Techniques

Oncoplastic volume displacement remains the preferred first-line reconstructive approach for many patients undergoing BCT, particularly those with moderate to large breasts and favorable tumor location [61,62]. By rearranging remaining breast tissue to fill the lumpectomy defect, these techniques preserve native tissue characteristics and avoid the introduction of foreign material or grafted fat into irradiated fields.

Patients with larger breast volume, sufficient tissue redundancy, and lower degrees of baseline asymmetry are generally best suited for volume displacement approaches [63,64]. Tumors located in the upper pole or lateral breast are often more amenable to these techniques, whereas lower pole and central defects may pose greater challenges [29,65]. Radiation exposure remains a critical modifier of outcomes; although oncoplastic rearrangement can be performed in irradiated tissue, fibrosis and volume contraction may compromise long-term symmetry [66].

Risk information is particularly important when considering contralateral symmetrization. While bilateral procedures can improve symmetry, they increase operative complexity and may not align with all patients’ preferences or risk tolerance [67,68]. Personalized counseling should address the likelihood of delayed asymmetry due to radiation-related changes and the potential need for future revision even after initially successful oncoplastic reconstruction [23].

### 5.2. Oncoplastic Volume Replacement Techniques

Volume replacement techniques represent an essential component of personalized reconstruction after breast-conserving therapy and are particularly valuable when lumpectomy defects cannot be adequately managed with parenchymal rearrangement alone. In contrast to volume displacement, which relies on redistribution of residual breast tissue, volume replacement introduces vascularized tissue from outside the breast to restore contour and minimize deformity. This approach is especially relevant in patients with small- to moderate-sized breasts, limited tissue redundancy, higher tumor-to-breast volume ratios, or defects in anatomically challenging locations where volume displacement may produce unacceptable distortion or require excessive contralateral symmetrization. Chest wall perforator flaps, including LICAP, TDAP, and related lateral thoracic perforator-based flaps, as well as mini-latissimus dorsi flaps, have emerged as useful options in this setting. These techniques can expand eligibility for breast conservation while preserving acceptable oncologic and aesthetic outcomes [69,70,71].

From a personalization standpoint, volume replacement is often best suited to patients in whom preservation of breast shape is unlikely with tissue rearrangement alone, but who do not require or desire more extensive total breast reconstruction. Flap selection depends on defect location, breast size and shape, donor site availability, prior scars, and surgeon expertise. LICAP and related lateral chest wall perforator flaps are particularly useful for lateral breast defects, whereas other perforator or pedicled regional flaps may better address lower pole, central, or more extensive volume deficits. Mini-latissimus dorsi flaps remain a useful option when a larger volume of well-vascularized tissue is needed, although this comes at the cost of greater donor-site morbidity than perforator-based options [70,72,73].

These techniques also have relevance in the post-radiation setting. Because volume replacement introduces healthy vascularized tissue into a previously treated field, it may offer advantages in selected patients with localized radiation-related contour deformity or tissue deficiency, although outcomes remain dependent on defect severity, tissue quality, and timing of intervention. Available literature suggests that chest wall perforator and other locoregional flap approaches can achieve favorable aesthetic outcomes with acceptable complication profiles, including in patients receiving adjuvant radiotherapy, but careful counseling remains essential because fibrosis, asymmetry, and the potential need for revision may still evolve over time [72,74].

Within a risk-informed framework, volume replacement should be viewed as complementary to volume displacement rather than as a secondary alternative. Patients with favorable breast size and tissue redundancy may still be best served by displacement alone, whereas those with limited native tissue, unfavorable defect geometry, or a desire to avoid more extensive whole-breast reconstruction may derive particular benefit from regional flap-based replacement. Incorporating these options into preoperative counseling broadens the reconstructive algorithm and improves alignment between defect characteristics, tissue biology, and patient goals.

### 5.3. Implant-Based Augmentation After Lumpectomy

Implant-based augmentation following lumpectomy represents a distinct and under-discussed reconstructive pathway. This approach may be particularly appealing to patients with small breast volume, limited tissue redundancy, or pre-existing augmentation who desire restoration of volume rather than tissue rearrangement [17,75]. However, the introduction of implants into a partially irradiated breast fundamentally alters the risk profile and demands careful patient selection.

Radiation exposure is the dominant determinant of outcomes in this setting. Even when radiation is delivered in a partial or tangential field, capsular contracture, implant malposition, and aesthetic distortion are more common than in nonirradiated breasts [76]. The plane of implant placement further modulates risk. Subglandular placement may exacerbate visible contour irregularities in thin or fibrotic tissue, whereas subpectoral or prepectoral placement introduces trade-offs related to animation deformity, implant coverage, and interaction with irradiated skin [77,78,79,80,81].

Patients with prior breast augmentation constitute a unique subgroup. In these individuals, implant exchange or augmentation after lumpectomy may appear straightforward but carries heightened risk of contracture and asymmetry when radiation is added to a previously unirradiated implant pocket [51,82]. Risk information should incorporate not only implant history but also patient expectations regarding long-term implant maintenance and tolerance for potential explantation or conversion to alternative reconstructive strategies.

Adjunctive materials such as acellular dermal matrices (ADM) or synthetic meshes have also been explored to improve implant support and soft-tissue coverage in selected cases of implant-based reconstruction following breast conservation. These materials may help reinforce thin or compromised tissues, particularly in patients with limited native breast parenchyma or prior radiation exposure [83]. However, evidence supporting ADM use in the post-lumpectomy reconstruction setting remains limited compared with the mastectomy reconstruction literature, and careful patient selection remains essential.

Given these considerations, implant-based augmentation after BCT may be most appropriate for carefully selected patients with limited radiation exposure, favorable tissue quality, and a clear understanding of the likelihood of future revision. In this context, revision burden, rather than short term surgical complications, often represents the most meaningful outcome for patients.

### 5.4. Fat Grafting as Volume Replacement and Tissue Modulation

Autologous fat grafting occupies a central role in personalized reconstruction after BCT, offering both volume replacement and potential mitigation of radiation-induced tissue injury [56,84,85]. Its versatility allows it to be used as a primary reconstructive modality for small defects, an adjunct to oncoplastic rearrangement, or a salvage technique following implant-based reconstruction [84,86]. Patient selection is critical. Fat grafting is best suited for individuals with sufficient donor sites, realistic expectations regarding achievable volume, and willingness to undergo staged procedures [11]. In irradiated breasts, graft take may be unpredictable, and multiple sessions are often required [11,87]. While concerns regarding oncologic safety have diminished with accumulating evidence, ongoing surveillance considerations and patient anxiety may still influence acceptance of this approach [88,89]. However, large matched-cohort analyses further support the oncologic safety of autologous fat grafting. In a single-center matched case–control study evaluating more than 1000 postmastectomy breast reconstructions, the addition of fat grafting was not associated with increased rates of locoregional recurrence or decreased disease-free survival compared with matched controls [90].

Emerging literature has also described structured, protocol-based approaches to autologous fat grafting that frame reconstruction as a staged, iterative process [91,92]. Prospective studies utilizing standardized grafting protocols have demonstrated that predictable reconstruction can be achieved through sequential fat transfer sessions with controlled graft volumes and defined treatment intervals. These protocols also highlight the differential behavior of irradiated and non-irradiated tissues, with irradiated breasts often requiring additional sessions and lower per-session graft volumes to optimize graft retention and minimize complications. Such findings reinforce the concept that fat grafting reconstruction is best conceptualized as a longitudinal reconstructive pathway rather than a single operative intervention.

Investigational regenerative strategies have also been explored to enhance fat graft retention and mitigate radiation-induced tissue injury. Techniques incorporating platelet-rich plasma (PRP) or stromal vascular fraction (SVF) enrichment aim to improve graft survival through enhanced angiogenesis and regenerative signaling. Early clinical studies suggest potential benefits in irradiated tissue environments; however, these approaches remain experimental and lack robust long-term clinical validation [93]. Further prospective studies are needed to determine whether biologically augmented fat grafting can meaningfully improve reconstructive durability in patients treated with radiation.

From a personalization standpoint, fat grafting offers granular control over reconstructive intensity [94]. Small-volume grafting may address contour irregularities with minimal morbidity, while larger-volume reconstruction increases the likelihood of fat necrosis, calcifications, and need for revision [95]. Counseling should emphasize that fat grafting is frequently iterative and that aesthetic stability may evolve over time as radiation effects continue to unfold [56,87].

### 5.5. Autologous Reconstruction in Select Post-BCT Patients

Although frequently considered in salvage settings after failure of other reconstructive strategies, autologous tissue reconstruction may also represent an appropriate primary approach in selected patients with severe radiation-related deformity, substantial volume loss, or tissue environments unlikely to tolerate implant-based or fat grafting-based reconstruction [96]. These cases often represent salvage scenarios in which local tissue options have been exhausted or are no longer viable [96]. Risk information is essential when considering autologous reconstruction in this population. Patients must be evaluated for donor site suitability, overall medical fitness, and willingness to undergo more extensive surgery. While autologous tissue may offer improved durability and aesthetic outcomes in heavily irradiated fields, the morbidity of flap-based reconstruction must be weighed against patient goals and expectations [41,59].

### 5.6. Integrating Risk Information into Technique Selection

Rather than viewing reconstructive options as competing alternatives, a personalized approach frames them as complementary tools that can be sequenced or combined based on evolving risk profiles. Patients with low-risk features may achieve durable results with oncoplastic rearrangement alone, whereas higher-risk individuals may require staged fat grafting, implant augmentation with careful counseling, or eventual conversion to autologous reconstruction [97]. This risk-informed paradigm emphasizes adaptability over rigidity. As tissue characteristics and patient priorities change over time, reconstructive strategy should be revisited, reinforcing the importance of longitudinal planning and shared decision-making in reconstruction after BCT.

## 6. Personalized Decision-Making After Breast-Conserving Therapy: Timing of Reconstruction

The timing of reconstruction following breast-conserving therapy (BCT) is a critical and highly individualized decision that significantly influences both aesthetic outcomes and complication risk (Table 2) [44,98]. Unlike mastectomy reconstruction, where immediate reconstruction is often encouraged to preserve the skin envelope and streamline care, reconstruction after BCT must account for the evolving biological effects of radiation, oncologic treatment sequencing, and patient readiness for additional surgery [44].

### 6.1. Immediate Versus Delayed Reconstruction

Immediate reconstruction at the time of lumpectomy or shortly thereafter may be appropriate for select patients with favorable risk profiles, limited anticipated radiation effects, and clear reconstructive goals [98]. Oncoplastic volume displacement techniques are most employed in this setting, as they allow defect correction using native tissue before radiation-induced fibrosis becomes established [14,99,100]. For appropriately selected patients, immediate reconstruction may reduce the need for later interventions and provide early restoration of breast contour [100,101]. However, early intervention carries the risk of underestimating the extent of radiation-related changes that may emerge over time. Volume loss, skin contraction, and asymmetry may progress months to years after treatment, potentially diminishing the durability of immediate reconstructive results [19]. For patients with higher-risk features, such as extensive radiation fields, smaller breast volume, or medical comorbidities, delayed reconstruction may offer a more predictable foundation for long-term outcomes [44,102].

### 6.2. Reconstruction in the Post-Radiation Setting

Delayed reconstruction allows surgeons to assess the stable effects of radiation before intervening, facilitating more accurate planning and patient counseling [44]. In this context, reconstructive techniques such as fat grafting or implant-based augmentation can be tailored to the established tissue environment. However, delayed intervention must contend with fibrosis, reduced tissue compliance, and compromised vascularity, all of which increase the likelihood of staged procedures and revision surgery. Personalization is particularly important when considering delayed implant-based reconstruction. While some patients may tolerate implant placement in a radiated breast with acceptable outcomes, others may experience progressive capsular contracture or aesthetic distortion over time [44,103,104]. Incorporating patient-specific risk factors into timing decisions helps align reconstructive strategy with long-term expectations and tolerance for revision [97]. Emerging radiation delivery strategies may also influence reconstructive decision-making. Intraoperative radiation therapy (IORT), which delivers a focused dose of radiation at the time of lumpectomy, has been explored as an alternative to conventional whole-breast radiation in selected patients. By limiting radiation exposure to surrounding tissues, IORT may theoretically preserve tissue quality and expand opportunities for immediate reconstructive interventions. Early studies suggest that selected patients treated with IORT may experience reduced radiation-related fibrosis and improved reconstructive predictability; however, long-term oncologic and reconstructive outcomes remain under investigation [105,106].

### 6.3. Staged and Sequential Reconstruction

Many reconstructive pathways after BCT are inherently staged, reflecting both biologic realities and patient preferences. Fat grafting is often performed in multiple sessions to gradually restore volume and improve tissue quality [56,87]. Staging allows for incremental assessment of graft take, contour improvement, and patient satisfaction while minimizing morbidity [107]. Sequential approaches may also involve escalation of reconstructive intensity over time. Patients initially managed with conservative interventions may later pursue more extensive reconstruction as tissue characteristics stabilize or aesthetic priorities evolve [27]. This adaptability underscores the importance of framing reconstruction after BCT as a longitudinal process rather than a single operative event.

Ultimately, the optimal timing of reconstruction after BCT must integrate oncologic considerations, tissue biology, and patient-centered goals [44,108]. Transparent discussion of the trade-offs associated with early versus delayed intervention, including the likelihood of revision, durability of results, and impact on quality of life, is essential to personalized decision-making [108]. By tailoring timing to individual risk profiles and expectations, surgeons can better align reconstructive planning with outcomes that matter most to patients [108].

## 7. Risk Prediction, Patient Counseling, and Shared Decision-Making in Personalized Reconstruction

Personalized reconstruction after breast-conserving therapy (BCT) depends not only on technical execution but on accurate risk prediction and effective patient counseling. Given the heterogeneity of tissue characteristics, treatment history, and patient priorities in this population, shared decision-making must be grounded in transparent discussion of both short-term risks and long-term reconstructive trajectories [109,110].

### 7.1. Limitations of Existing Risk Prediction Tools

Existing risk prediction models in breast reconstruction have largely been developed in the context of mastectomy-based reconstruction and do not adequately account for the unique variables encountered after BCT [111,112,113]. Factors such as nonuniform radiation fields, partial volume loss, and evolving tissue fibrosis are difficult to capture in traditional calculators that focus on binary outcomes such as infection or implant loss [112,114]. As a result, these tools may underestimate the likelihood of delayed complications or revision surgery in the post-BCT population.

Moreover, many commonly reported outcomes fail to reflect patient priorities. Complication rates reported at 30 or 90 days do not capture longitudinal changes in breast contour, progressive asymmetry, or the cumulative burden of staged procedures [112,115,116]. This mismatch between measured outcomes and lived patient experience limits the utility of existing models for counseling patients considering reconstruction after BCT.

### 7.2. Incorporating Revision Burden into Counseling

For patients undergoing reconstruction after BCT, the probability and frequency of revision procedures represent clinically meaningful outcomes that should be incorporated into preoperative counseling [27]. Techniques such as fat grafting and implant-based augmentation often require multiple interventions to achieve durable results, particularly in irradiated tissue [11,44,56,87]. Framing reconstruction as a process rather than a single operation helps set realistic expectations and allows patients to weigh aesthetic benefits against the time, recovery, and emotional investment associated with staged care.

Personalized counseling should explicitly address how individual risk factors, such as radiation exposure, breast size, and comorbidities, influence the likelihood of revision. By normalizing the need for secondary procedures in appropriate contexts, surgeons can reduce dissatisfaction and enhance alignment between patient expectations and surgical outcomes [117].

### 7.3. Shared Decision-Making as a Core Component of Personalization

Shared decision-making is central to personalized reconstruction after BCT. Patients bring diverse values and priorities to reconstructive planning, including tolerance for asymmetry, willingness to undergo multiple procedures, and preferences regarding implants or autologous tissue [118]. These considerations often carry equal weight to physiologic risk factors and must be integrated into reconstructive recommendations [118]. Effective shared decision-making requires clear communication of uncertainty, particularly in scenarios where long-term outcomes are difficult to predict [119,120]. Visual aids, decision trees, and examples of anticipated reconstructive trajectories may help patients contextualize risk and make informed choices that align with their goals [109,121,122].

Practice patterns in breast reconstruction also vary considerably across geographic regions. International surveys, including data from the European Society of Plastic, Reconstructive and Aesthetic Surgery (ESPRAS), demonstrate substantial differences in reconstructive preferences, radiotherapy management strategies, and guideline implementation between European and North American surgeons. These findings underscore the ongoing need for more standardized, evidence-based frameworks to guide reconstructive decision-making while still allowing for individualized patient-centered care [123].

### 7.4. Patient Reported Outcomes

Patient-reported outcomes are increasingly recognized as essential measures of success in breast reconstruction. Validated instruments such as the BREAST-Q have been widely used to evaluate domains including satisfaction with breasts, psychosocial well-being, physical well-being, and sexual well-being following breast surgery [124]. Although originally developed for mastectomy and reconstruction populations, BREAST-Q modules have also been applied to patients undergoing breast-conserving therapy to assess aesthetic satisfaction and quality-of-life outcomes. Additional tools, including breast cancer-specific quality-of-life instruments such as the EORTC QLQ-BR23 and related modules, have also been used to capture patient perspectives following breast-conserving treatment. Incorporating standardized PRO instruments into reconstructive research is critical for evaluating how surgical strategies influence long-term patient satisfaction and quality of life.

Future efforts should focus on developing predictive models that incorporate radiation characteristics, breast morphology, and patient-reported outcomes alongside traditional complication metrics. Institutional data and multicenter registries may play a critical role in refining these tools and validating their applicability to diverse patient populations [125,126]. By integrating risk prediction with patient-centered counseling and shared decision-making, personalized reconstruction after BCT can move beyond technique-driven algorithms toward care pathways that prioritize durability, satisfaction, and alignment with individual patient goals.

## 8. Future Directions

As the population of patients treated with breast-conserving therapy (BCT) continues to grow, there is an increasing need for reconstructive paradigms that move beyond technique-driven decision-making toward truly personalized care. Several emerging areas offer opportunities to improve risk prediction, patient selection, and long-term outcomes in this population.

First, the development of BCT-specific predictive models represents a critical unmet need. Existing reconstruction risk calculators do not adequately incorporate the heterogeneity of radiation fields, partial-volume tissue loss, or longitudinal changes in breast morphology that characterize post-BCT patients [125,126,127,128,129]. Future models should integrate treatment-specific variables, such as radiation dose distribution and timing, with patient-level factors and reconstructive technique to more accurately predict complications, revision burden, and patient-reported outcomes.

Second, advances in imaging and intraoperative assessment may enhance personalization of reconstructive planning. Preoperative imaging techniques that characterize tissue quality and vascularity, as well as intraoperative tools that assess perfusion, could help refine patient selection and guide technique choice in compromised tissue environments [130,131,132,133]. These technologies may be particularly valuable in identifying patients who are unlikely to tolerate implant-based reconstruction or who may benefit from staged fat grafting or autologous approaches.

Third, incorporation of patient-reported outcomes into both clinical practice and research is essential to advancing personalized reconstruction after BCT. Instruments that capture satisfaction, body image, and quality of life over time can provide insights into how reconstructive strategies align with patient priorities and may help identify subgroups that benefit most from specific approaches [134,135]. Embedding these measures into registries and longitudinal studies will be critical for refining shared decision-making frameworks.

Importantly, the conceptual framework proposed in this review has not yet been prospectively validated and many current reconstructive strategies in the post-BCT setting are supported primarily by retrospective cohort data and institutional experience, underscoring the need for prospective comparative studies to refine evidence-based treatment pathways. Future research should focus on developing and testing predictive models that incorporate variables unique to the post-breast-conserving therapy population, including radiation field characteristics, breast morphology, tumor location, and patient-reported outcome measures. Prospective multicenter registries and longitudinal cohort studies will be particularly valuable for evaluating how these factors interact to influence complication rates, revision burden, and long-term aesthetic outcomes. Such data will be essential to refine and validate risk-informed reconstructive algorithms.

Finally, future research should emphasize longitudinal follow-up and durability of outcomes [8,104,134]. Reconstruction after BCT is often an evolving process, and short-term metrics fail to capture the full patient experience [8,19]. Studies that assess outcomes over years rather than months will be essential to understanding how personalized strategies perform over time and to informing evidence-based counseling.

An important limitation of the current literature informing reconstructive strategies after breast-conserving therapy is the predominance of retrospective, single-institution studies with relatively small sample sizes. These studies often involve heterogeneous patient populations, variable radiation protocols, and differing reconstructive techniques, which can limit the generalizability of findings and make direct comparisons between approaches difficult. High-quality prospective data specifically addressing reconstruction in the post-BCT population remain limited. Future research efforts should prioritize multicenter prospective studies, standardized reporting of complications and revision burden, and incorporation of patient-reported outcomes to better define evidence-based reconstructive pathways.

## 9. Conclusions

Reconstruction after breast-conserving therapy occupies a distinct and under-recognized space within breast reconstruction, characterized by heterogeneous tissue environments, evolving radiation effects, and highly individualized patient goals. A personalized, risk-informed approach offers an effective framework for reconstructive planning after breast-conserving therapy. By integrating patient-specific risk factors, treatment history, and psychosocial preferences, surgeons can tailor technique selection and timing to optimize durability, minimize revision burden, and align outcomes with patient expectations. Importantly, meaningful outcomes in this setting extend beyond short-term complications to include long-term aesthetic stability and patient-reported satisfaction. By embracing personalization as a guiding principle, reconstructive strategies after breast-conserving therapy can best align with the diverse needs and priorities of patients navigating survivorship.

## Figures and Tables

**Table 1:** Patient factors influencing personalized reconstruction after breast-conserving therapy.

Risk Factor Category	Clinical Considerations	Reconstructive Implications	Preferred Reconstructive Approach
Breast size and tissue redundancy	Larger breasts with sufficient parenchyma may tolerate tissue rearrangement; smaller breasts have limited reserve	Influences feasibility of volume displacement	Larger breasts: oncoplastic volume displacement; smaller breasts: volume replacement flap, implant augmentation, or staged fat grafting
Radiation exposure	Degree and distribution of radiation-related fibrosis affect tissue compliance and vascularity	Higher risk of complications and revision	Staged fat grafting, cautious implant use, consideration of regional flap or autologous reconstruction in severe deformity
Tumor location	Lower pole or central tumors often create more visible deformity	May limit effectiveness of simple rearrangement	Volume replacement flaps, fat grafting, or combined oncoplastic techniques
Prior breast surgery	Previous augmentation, reduction, or lumpectomy alters tissue planes and implant dynamics	Increased risk of capsular contracture or asymmetry	Implant exchange with caution, fat grafting, or regional flap reconstruction
Patient comorbidities	Smoking, obesity, diabetes impair wound healing	Increased complication risk	Conservative approaches such as staged fat grafting or limited oncoplastic correction
Patient preferences	Willingness to undergo staged procedures or accept asymmetry	Determines acceptable reconstructive pathway	Minimal intervention, staged fat grafting, implant augmentation, or autologous reconstruction depending on goals

**Table 2:** Personalized timing strategies for reconstruction after breast-conserving therapy.

Reconstruction Timing	Advantages	Limitations	Best Candidates
Immediate	Early contour restoration	Radiation effects may evolve	Low-risk, favorable anatomy
Delayed	Stable tissue assessment	Fibrosis, staged care	Higher-risk, irradiated tissue
Staged	Adaptable, incremental	Multiple procedures	Patients tolerant of revisions

## Data Availability

No new data were created or analyzed in this study. Data sharing is not applicable to this article.
